# The effect of acyl-CoA synthetase long-chain family member 5 on triglyceride synthesis in bovine preadipocytes

**DOI:** 10.5194/aab-62-257-2019

**Published:** 2019-05-06

**Authors:** Xiang Yu, Xibi Fang, Hang Xiao, Zhihui Zhao, Steffen Maak, Mengyan Wang, Runjun Yang

**Affiliations:** 1 College of Animal Science, Jilin University, 5333 Xi'an Road, Changchun 130062, P. R. China; 2 College of Agriculture, Guangdong Ocean University, Zhanjiang, 524088, P. R. China; 3 Institute of Muscle Biology and Growth, Leibniz Institute for Farm Animal Biology (FBN), Dummerstorf, 18196, Germany

## Abstract

Acyl-CoA synthetase long-chain family member 5 (*ACSL5*) is a member of the acyl coenzyme A (CoA) long-chain synthase families (ACSLs), and it plays a key role in fatty acid metabolism. In this study, we proved an association between the *ACSL5* gene and triglyceride metabolism at the cellular level in cattle. pBI-CMV3-*ACSL5* and pGPU6/GFP/Neo-*ACSL5* plasmids were constructed and transfected into bovine preadipocytes by electroporation. The expression level of *ACSL5* was detected by real-time quantitative PCR and western blot. The triglyceride content was detected by a triglyceride kit. The results indicated that the expression level of *ACSL5* mRNA and protein in the pBI-CMV3-*ACSL5*-transfected group was significantly increased compared with those in the control group. Furthermore, the pGPU6/GFP/Neo-*ACSL5*-transfected group was significantly decreased compared with those in the control group. A cell triglyceride test showed that overexpression or silencing of the *ACSL5* gene could affect synthesis of cellular triglycerides. This study investigated the mechanism of ACSL on bovine fat deposition, and also provides a new candidate gene for meat quality traits in beef cattle.

## 1 Introduction

Lipid is a substance of biological origin that is soluble in nonpolar solvents in biology (Argetsinger and Carter-Su, 1996). And it not only acts as a structural component of cell membranes but also participates in energy conversion, information identification and delivery, material transport, and many other biological processes (Bowman et al., 2016; Bronkhorst et al., 2014). Mammalian cells contain diverse lipids, such as phospholipids, sphingolipids, cholesterol, and triglycerides. Meat quality and flavor mainly depend on the deposition of fat, and the most important factor is intramuscular fat deposition. Studies proved that intramuscular fat deposition mainly referred to triglyceride metabolism (Catala-Rabasa et al., 2011).

Triglycerides are the main constituents of body fat in farm animals and other mammals. Triglycerides are a kind of ester derived from glycerol and fatty acids after a dehydration condensation reaction. Triglyceride sources include endogenous synthesis and exogenous absorption, and exogenous triglycerides firstly decompose into fatty acids and glycerol rather than being deposited directly in adipocytes. Triglycerides that synthesize and deposit in adipocytes need a series of enzymatic reactions to be involved with multiple genes, such as acetyl-CoA, acyl-CoA, and acetyl-CoA carboxylase. In addition, the metabolic rate of triglycerides also affects the deposition of intramuscular fat. Therefore, fat deposition is regulated by the synthesis and decomposition of triglycerides and it is the result of the coordinated regulation of cellular, hormonal, and genetic levels (Catala-Rabasa et al., 2011).

Acyl-CoA synthetase long-chain families (ACSLs) are a class of enzymes that include 26 members and have tissue and substrate specificity (Fahy et al., 2011, 2009), and they are key enzymes that regulate the import/export system in the absorption of fatty acids into cells. ACSLs are a member of acyl-CoA synthetase families (ACSs) that are indispensable in the activation of fatty acids, and play a key role in mammalian metabolism of fatty acids (Nelson and Ackman, 1988). As the carbon chain length of most foodborne fatty acids is 10–20 carbon atoms, ACSL is the most important member of the ACSs and shares a number of key features (Fahy et al., 2011; Gulick et al., 2003). Different ACSL members have similar functional protein structural units and they all have corresponding AMP (adenosine monophosphate) binding sites and CoA (coenzyme A) binding sites. Previous works found that *ACSL5* is a critical activator of dietary long-chain fatty acids and can decrease fatty acid activation in human jejunum (Meller et al., 2013). Bu et al. (2010) conducted a series of trials in which knockdown *ACSL5* could decrease hepatic secretion in neutral lipid synthesis (Bu and Mashek, 2010). The activity of *ACSL5* could affect intestinal microbial relationships and affect lipid metabolism further (Sheng et al., 2018). Overall, the above studies indicate that *ACSL5* plays an important role in the fat metabolism pathway of mammalian animals.

Our previous studies have also shown that the mutations of the *ACSL5* gene in the Simmental cattle population were associated with carcass composition and fat deposition traits of beef cattle (Xiao et al., 2016). However, the function of *ACSL5* on triglyceride synthesis in bovine preadipocytes is rarely reported. To study the function of *ACSL5*, the overexpression and RNA interference vector of *ACSL5* were constructed, and the transfection of bovine preadipocytes was performed by electroporation. Then, intracellular triglyceride contents were detected in preadipocytes of different groups. The results of the present study lay the foundation for further exploring mechanisms of ACSL on bovine fat deposition and also provide a new candidate gene for meat quality traits in beef cattle.

## 2 Material and methods

### 2.1 Ethics statements

Animal experiments strictly abided with the “Guide for the Care and Use of Laboratory Animals” by the Animal Health and Use Committee of Jilin University (permit number: SYXK (Ji) pzpx20181227083).

### 2.2 Relative mRNA expression level of the *ACSL5* gene in bovine tissues

This study involved three Chinese Simmental steers (28 months old) from the Center for Laboratory Animal Science of Inner Mongolian University. Various tissues, including liver, spleen, kidney, visceral fat, subcutaneous fat, and longissimus dorsi, were selected to detect the expression of the *ACSL5* gene. Total RNA was extracted from tissues using TRIzol reagent (Invitrogen, USA) and was checked with agarose gel electrophoresis. The concentration was measured using a spectrophotometer. Then, 1 µg RNA was reversely transcribed into cDNA using a Prime Script^™^ RT reagent kit with gDNA Eraser (Takara, Dalian, China) following the manufacturer's protocol. A pair of specific primers, *ACSL5*-F: 5${}^{\prime }$-CCCTACAGATGGCTGTCCTAC-3${}^{\prime }$ and *ACSL5*-R: 5${}^{\prime }$-GCTCCCAAGGTGTCATACAAG-3${}^{\prime }$ were designed using Primer Premier 5.0, and qRT-PCR (quantitative reverse transcription PCR) was carried out using SYBR^®^ Premix Ex taq^™^ (Takara, Dalian, China). The following PCR amplification conditions were used: 95 ${}^{\circ }$C for 5 min; 40 cycles of 95 ${}^{\circ }$C for 30 s and 60 ${}^{\circ }$C for 30 s.

### 2.3 Construction of overexpression vector pBI-CMV3-*ACSL5*

The nested PCR outer primers (O-*ACSL5*-F: 5${}^{\prime }$-TGAGGACTCGCAGCAATTCAT-3${}^{\prime }$; O-*ACSL5*-R: 5${}^{\prime }$-TGAAGGCGGGTAAGGTACAGC-3${}^{\prime }$) were designed according to the regions of the 3${}^{\prime }$ UTR (untranslation region) and the 5${}^{\prime }$ UTR of *ACSL5* gene. The inner primers (the sequence of upstream primer: 5${}^{\prime }$-GGCCCGCTAGCATGCTTTTTATCT TTAACT-3${}^{\prime }$; the sequence of downstream primer: 5${}^{\prime }$-GAATCATCGATCTACTCCTGGGTGTTCT C-3${}^{\prime }$) were designed based on the start codon and stop codon of the gene. The first nested PCR amplification was performed using the liver cDNA template. The first step amplification product was used as template for the second PCR. Then the resulting amplicon of approximately 2000 bp was purified and ligated into a pMD18-T simple vector (Takara, Dalian, China). The ligation product was digested with Nhel (NEB, UK) and ClaI (NEB, UK) and ligated into the expression vector pBI-CMV3 (Clontech, USA) that digested with the same enzymes. At the same time, the pBI-CMV3-*ACSL5* plasmid was identified by sequencing (Shanghai Sangon Biotech).

### 2.4 Construction of interference vector pGPU6/GFP/Neo-*ACSL5*

Four sequence-specific single-stranded target sequences were designed according to the sequence of bovine *ACSL5* mRNA and the design principle of shRNA. Then the annealed oligonucleotides were cloned into interference vector pGPU6/GFP-Neo by Shanghai Gemma Pharmaceutical Technology Co., Ltd. The pGPU6/GFP-Neo-*ACSL5* was transfected into bovine fibroblasts to study the quantity of fluorescent expression. Compared with that of the control group, shRNA2 (the transcription sequence: 5${}^{\prime }$-GATATCGCCATGGTAATCTGTTTCAAGAGAACAGAT TACCATGG CGATATCTT-3${}^{\prime }$) had the highest efficiency of RNA interference.

### 2.5 Cultivation and identification of bovine preadipocytes

Adipose tissue from the kidneys was collected under aseptic operation and transported to the laboratory. Primary cells were obtained from tissues by enzymatic digestion. The digested cells were cultured in DMEM/F12 (Dulbecco's Modified Eagle Medium and Nutrient Mixture F-12, HyClone, USA) medium containing 10 % FBS (fetal bovine serum; Invitrogen, USA). Each 50 mL of maintenance medium was supplemented with 500 µg of insulin (Abcam, UK), 1.65 µmol of calcium pantothenate (Sigma, USA), and 8.5 µmol of bovine transferrin (Sigma, USA). After 7 d of induced differentiation, cells were stained with Oil Red O (Sigma, USA) and observed under the microscope (Olympus, CKX41, Japan) until the round and bright lipid droplets had formed in the cytoplasm. Total RNA was extracted from bovine preadipocytes using TRIzol reagent. One microgram total RNA was reverse transcribed into cDNA to study the effect of expression of *ACSL5* gene on the triglycerides content in preadipocytes.

### 2.6 Transfection of bovine preadipocytes

The cells were seeded into six-well plates (Falcon, Franklin Lake, NJ, USA) in DMEM/F12 with 10 % FBS and 1 % penicillin/streptomycin (HyClone, USA). Bovine fetal fibroblasts were transfected with FuGENE^®^ HD Transfection Reagent (Promega, USA) according to the manufacturer's instruction. Preadipocytes were transfected by electroporation. Briefly, 300 µL of electroporation buffer (Opti-MEM, Gibco, USA) and 30 µg of the vectors were gently and evenly added to the electroporation cuvette (BTX, USA). Then the preadipocytes were transfected by electroporation under a pulse parameter of 3 pulses per millisecond and a voltage parameter of 200 V. After 24–48 h cultivation, the expression of green fluorescent protein (GFP) in fibroblast cells was observed under a fluorescence microscope (Nikon TE2000, Japan), and the preadipocytes were cultured for 36–72 h after transfection. The overexpression groups included a pBI-CMV3-*ACSL5* group and a pBI-CMV3 control group. The RNA interference groups included a pGPU6/GFP/Neo-*ACSL5* group and a pGPU6/GFP/Neo control group.

### 2.7 Analysis of *ACSL5* mRNA expression levels by quantitative RT-PCR

To investigate cellular *ACSL5* mRNA levels, the cells were harvested and lysed after transfection for 36 h. Total RNA was extracted and reverse transcribed into cDNA. Primers (*ACSL5*-F: 5${}^{\prime }$-CCCTACAGATGGCTGTCCTAC-3${}^{\prime }$ and *ACSL5*-R: 5'-GCTC CCAAGGTGTCATACAAG-3${}^{\prime }$) were designed and qRT-PCR was carried out using SYBR^®^ Premix Ex taq^™^. The bovine β-actin gene (ACTB-F: 5${}^{\prime }$-ATGCTTCTAGGC GGACTGTTA-3${}^{\prime }$ and ACTB-R: 5${}^{\prime }$-TGCCAATCTCATCTCGTTTTC-3${}^{\prime }$) was selected as the internal control gene.

### 2.8 Analysis of *ACSL5* protein expression levels by western blot

The cells were lysed using RIPA buffer (radio-immunoprecipitation assay lysis buffer, BOSTER, China) supplemented with protein phosphatase inhibitors after transfection for 48 h. Cell lysate was centrifuged at 12000×g for 5 min at 4 ${}^{\circ }$C and the supernatants were collected. The concentration of protein was determined with a modified BCA (bicinchoninic acid protein quantitation assay) protein assay (KeyGEN BioTECH, China) using a microplate spectrophotometer (BioTek Instruments EON, USA). Equal concentrations of various proteins were resolved by SDS-PAGE and then transferred onto PVDF (polyvinylidene fluoride) membranes (Bio-Red Laboratories, Inc., USA). After blocked with 1 % BSA (bovine serum albumin) for 2 h, the PVDF membrane was washed with TBST and incubated with rabbit polyclonal anti-*ACSL5* antibody (Abcam, USA) and β-actin (Abcam, USA) over night at 4 ${}^{\circ }$C. After being washed with TBST, the membrane was incubated with enhanced chemiluminescent HRP-conjugated (horseradish peroxidase-conjugated) anti-rabbit secondary antibody (BioWorld, USA) for 1.5 h at room temperature. The membrane was washed with TBST and the developer (Invitrogen, USA) was uniformly dropped on the membrane. The membrane was exposed using a chemiluminescence imager (Tanon, Shanghai, China) after 3 min of reaction. The gray values of protein bands were digitized by Image-Pro Plus.

### 2.9 Detection of the triglyceride content in preadipocytes

Cells transfected with different vectors in six-well culture plates were collected after transfection. The triglyceride contents were determined using a triglyceride kit (KeyGEN BioTECH, China) according to the manufacturer's protocol. The OD values at 550 nm of the sample were detected with a microplate reader (BioTek Instruments EON, USA), and the concentration of triglycerides was calculated according the standard curve. The cellular content of triglycerides was adjusted based on the quantity of protein.

### 2.10 Statistical analysis

The relative expression level of *ACSL5* gene was analyzed using the comparative Ct method ($2^{-\Delta \Delta \text{Ct}})$ and the significance was evaluated by t test. The data analysis was carried out with SPSS15.0. Statistically significant difference was defined as p<0.05. Experimental data were shown as the mean ± SE (standard error).

## 3 Results

### 3.1 The abundance of *ACSL5* in bovine tissues

**Figure 1:**
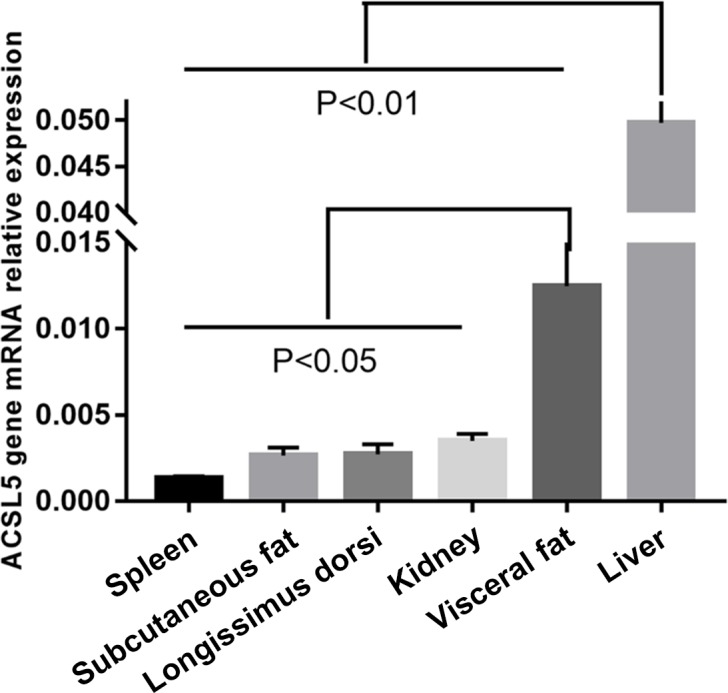
The abundance of *ACSL5* in six bovine tissues.

We investigated *ACSL5* mRNA expression level in different tissues of beef cattle. The highest expression level of *ACSL5* was in liver and had an extremely significant difference when comparing the other tissues (p<0.01). The second was in visceral fat and had a twofold higher expression than spleen, subcutaneous fat, longissimus dorsi, or kidney. The spleen showed the lowest level of *ACSL5* expression (Fig. 1). In summary, these results show that the expression of *ACSL5* may have an influence on subcutaneous fat metabolism and visceral fat deposition.

### 3.2 Construction of the overexpression and RNA interference vectors

**Figure 2:**
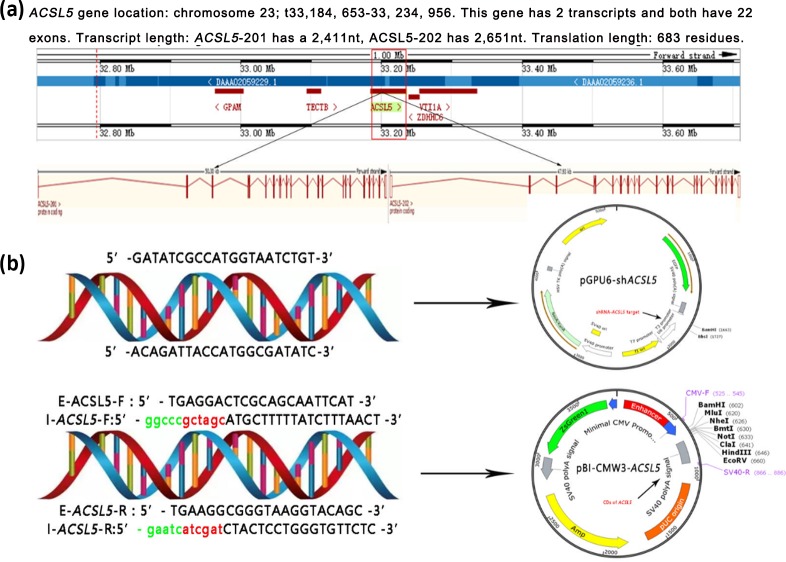
Gene mapping of bovine *ACSL5* and vectors incorporating the *ACSL5* gene. **(a)** Bovine *ACSL5* gene location and structure features. **(b)** pGPU6/GFP/Neo-ACSL and pBI-CMV3-*ACSL5*. E-*ACSL5*: external primers for nested PCR; I-*ACSL5*: internal primers for nested PCR (green indicates protective bases, red indicates restriction sites).

*ACSL5*, which contains 22 exons and 21 introns, is located on bovine chromosome 26 (33, 184, 653-33, 234, 956). *ACSL5* has two transcripts: one is 2651 nt in length, and the other is 2411 nt. The two transcripts only have a difference in the 5${}^{\prime }$ UTR region and both encode 683 amino acids (Fig. 2a). To reveal the effect of the *ACSL5* gene on the metabolism of triglycerides, the overexpression vector and RNA interference vector of the bovine *ACSL5* gene were constructed (Fig. 2b). Sequence analysis showed that the CDS (coding sequence) fragments of *ACSL5* were successfully inserted into the pBI-CMV3 vector mediated by restriction endonucleases NheI (GCTAGC) and ClaI (ATCGAT); the shDNA insert (siRNA target oligonucleotide sequences for *ACSL5* gene) was successfully cloned and framed into the multiple cloning sites of the pGPU6/GFP/Neo vector.

### 3.3 Identification of bovine preadipocytes differentiation

**Figure 3:**
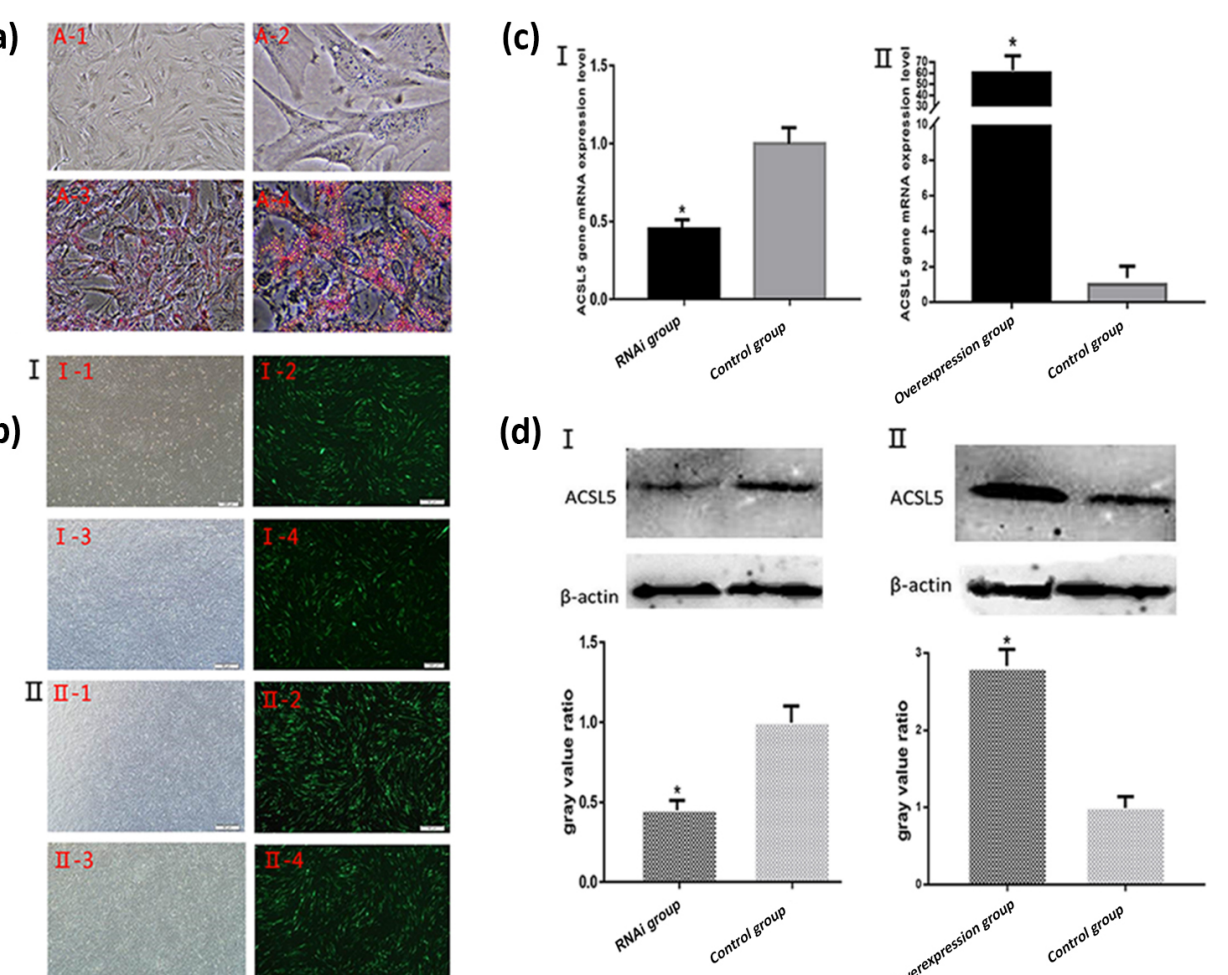
Oil Red O staining of bovine preadipocyte and fluorescence detection and the expression levels of *ACSL5* mRNA and protein. **(a)** A-1: Undifferentiated bovine preadipocytes (unstained, 200×); A-2: undifferentiated bovine preadipocytes (unstained, 400×); A-3: differentiated bovine preadipocytes (stained, 200×); A-4: differentiated bovine preadipocytes (stained, 400×). **(b)** Fluorescence detection of bovine preadipocytes transfected with shRNA-*ACSL5* and pBI-CMV3-*ACSL5*. I-1: shRNA-*ACSL5*-transfected bovine preadipocytes (visible light); I-2: shRNA-*ACSL5*-transfected bovine preadipocytes (fluorescence); I-3: negative control vector-transfected bovine preadipocytes (visible light); I-4: negative control vector-transfected bovine preadipocytes (fluorescence). II-1: pBI-CMV3-*ACSL5*-transfected bovine preadipocytes (visible light); II-2: pBI-CMV3-*ACSL5*-transfected bovine preadipocytes (fluorescence); II-3: negative control vector-transfected bovine preadipocytes (visible light); II-4: negative control vector-transfected bovine preadipocytes (fluorescence). **(c)** The expression level of *ACSL5* mRNA in bovine preadipocytes. c-I: the expression level of *ACSL5* mRNA in bovine preadipocytes transfected with the shRNA-*ACSL5* and pGPU6/GFP/Neo plasmids. c-II: the expression level of *ACSL5* mRNA in bovine preadipocytes transfected with pBI-CMV3-*ACSL5* and pBI-CMV plasmids. **(d)** The expression level and the gray value ratio of *ACSL5* protein in bovine preadipocytes. D-‡T: the expression level and the gray value ratio in bovine preadipocytes transfected with shRNA-*ACSL5* and pGPU6/GFP/Neo plasmids. D-‡U: expression level and the gray value ratio in bovine preadipocytes transfected with pBI-CMV3-*ACSL5* and pBI-CMV plasmids.

After the induction of preadipocytes for 8 d, the cells were prone to be round and bright. Identification of adipocyte differentiation showed that a large number of lipid droplets formed. The droplets were stained red by oil Red O (Fig. 3a). The result indicated that the bovine preadipocytes induced differentiation into mature adipocytes successfully.

### 3.4 Transfection of bovine preadipocytes by electroporation

After bovine preadipocytes were transfected with pGPU6/GFP/Neo-*ACSL5* and negative control vector for 48 h, the expression of green fluorescence could be observed in the preadipocytes transfected groups but not in the blank control group (Fig. 3b). The result showed that the *ACSL5* interference vector was successfully transfected and highly expressed in bovine preadipocytes. The same result could be obtained in the pBI-CMV3-*ACSL5* vector transfected groups (Fig. 3b).

### 3.5 Analysis of *ACSL5* expression in preadipocytes transfected with different plasmids

The qRT-PCR results revealed that the *ACSL5* mRNA expression level in pGPU6/GFP/Neo-*ACSL5*-transfected cells was significantly decreased compared with that in the pGPU6/GFP/Neo-transfected cells (p<0.05, Fig. 3c). On the other hand, the *ACSL5* mRNA expression level in the pBI-CMV3-*ACSL5*-transfected cells was significantly increased compared with that in the pBI-CMV3 transfected group (p<0.05, Fig. 3c).

The preadipocytes were harvested and lysed after transfection for 48 h to investigate cellular *ACSL5* protein expression levels. Total protein was extracted from transfected cells and the content of *ACSL5* protein was analyzed by western blot. The result showed that the expression level of *ACSL5* protein in the pGPU6/GFP/Neo-*ACSL5* transfected group was significantly decreased compared with that in the pGPU6/GFP/Neo transfected group (p<0.05, Fig. 3d). Furthermore, the protein level in the pBI-CMV3-*ACSL5* transfected group was significantly increased compared with that in the pBI-CMV3 transfected group (p<0.05, Fig. 3d).

### 3.6 The effect of *ACSL5* on triglyceride content in preadipocytes

**Figure 4:**
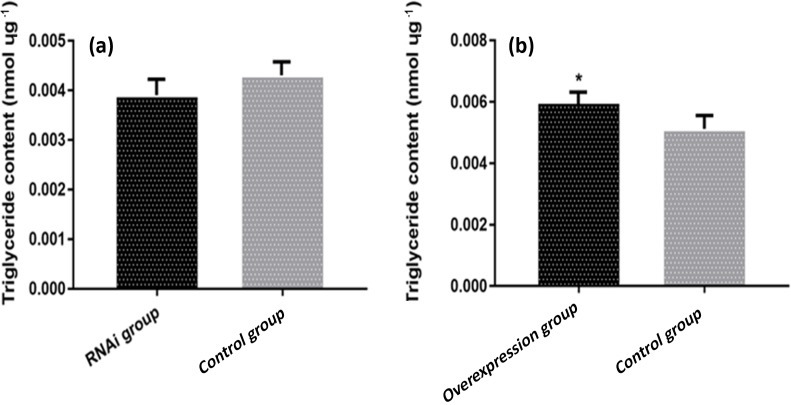
The content of triglyceride in preadipocytes of different transfection groups. **(a)** The triglyceride (TG) content of bovine preadipocytes transfected with the pBI-CMV3-*ACSL5* and pBI-CMV3 vectors. **(b)** The TG content of bovine preadipocytes transfected with the shRNA-*ACSL5* and pGPU6/GFP/Neo vectors.

The triglyceride content in preadipocytes of different transfection groups was analyzed. The result showed that the triglyceride content in the pBI-CMV3-*ACSL5* transfected group was significantly higher compared with that in the pBI-CMV3 transfected group (p<0.05). Meanwhile, compared with that in the pGPU6/GFP/Neo transfected group, the triglyceride content of bovine preadipocytes in the pGPU6/GFP/Neo-*ACSL5* transfection group was lower, but the difference did not achieve statistical significance (Fig. 4).

## 4 Discussion

ACSLs act on fatty acid activation in mammalian organisms, which is a prerequisite for fatty acids to participate in all physiological metabolic activities (Li et al., 2010). Anterior studies have noted the importance of the association between meat quality and ACSLs expression level. Bionaz and Loor (2008) have demonstrated that the sustained upregulation of ACSL1 enabled fatty acids toward copious milk fat synthesis in bovine mammary glands (Bionaz and Loor, 2008). After steers were fed with a high-starch diet, including ACSL1, some potential genes increased significantly (Graugnard et al., 2009). Compared to other ACSL family members, *ACSL5* is highly tissue specific. The expression of *ACSL5* has been associated with meat quality traits in beef cattle, which indicated that *ACSL5* might have a significant effect on triglyceride synthesis and lipid assimilation (Lopes-Marques et al., 2013). Our studies demonstrated the hypothesis that *ACSL5* could regulate triglyceride (TG, triacylglycerol) synthesis through cloning vectors and tranfections.

Eukaryotic expression vector is a common method for gene function verification by overexpressing genes. In the process of constructing the overexpression vector, there were two synonymous mutations, T264C and T702C, in the *ACSL5* gene coding region compared with the NCBI (National Center for Biotechnology Information) published sequence. After sequencing, we observed that all the cloned plasmids have the two mutation sites, indicating that T264C and T702C are most probably two SNP (single-nucleotide polymorphism) sites in wild cattle. In this experiment, mRNA expression level of *ACSL5* showed a significant increase in bovine preadipocytes transfected with pBI-CMV3-*ACSL5*. Although the mutation caused a triple-codon mutation, it did not change the amino acid type.

The shRNA interference is a common way to study the function of mammalian genes (Mashek et al., 2004; Bofill-De Ros and Gu, 2016). The position of the complementary sequence in shRNA has a great influence on the interference efficiency (Mendez et al., 1997; Shmakov et al., 2015; Snead et al., 2013). Therefore, we needed to conduct the screening of the interference sequence at the cellular level in the RNA interference experiment to confirm the interference sequence with the highest efficiency of interference. In this experiment, four shRNA interference vectors were designed. After screening, the vector with the highest interference effect was selected for electroporation. The present study was designed to determine whether *ACSL5* could impact triglyceride synthesis. In the overexpression transfection group, the content of TG was significantly increased. And the interference-transfection group was decreased but no significance. A possible explanation is that other members of the ACSLs family have compensatory mechanism effects on *ACSL5*.

Transfection of bovine preadipocytes with chemical methods has low transfection efficiency and cannot meet the experimental requirements (Watkins et al., 2007; Yang et al., 1996). Therefore, electroporation was used in our experiments. Electroporation, which can be used for a majority of cell transfections, is a physical cell transfection method (Zhang et al., 2004). The damage was negligible, and the transfection efficiency was satisfied.

In this experiment, the overexpression vector and interference vector were constructed and transfected into bovine preadipocytes successfully. In the overexpression transfection group, the content of triglyceride increased significantly compared with the control. And in the interference transfection group, the content of triglyceride decreased compared with control. These results all indicated that *ACSL5* could regulate triglyceride synthesis in bovine preadipocyte.

## 5 Conclusion

The study was to clarify the roles of *ACSL5* in regulating synthesis of triglycerides and lipid droplet formation in bovine preadipocytes. Research results confirmed that *ACSL5* gene could promote the synthesis of triglycerides in bovine adipocytes by participating in the fatty acid metabolism pathway. The interaction of *ACSL5* with related fatty acid synthase in the fatty acid biosynthesis pathway needs further analysis.

## Data Availability

The data sets are available upon request from the corresponding author.

## References

[bib1.bib1] Argetsinger LS, Carter-Su C (1996). Mechanism of signaling by growth hormone receptor. Physiol Rev.

[bib1.bib2] Bionaz M, Loor JJ (2008). ACSL1, AGPAT6, FABP3, LPIN1, and SLC27A6 are the most abundant isoforms in bovine mammary tissue and their expression is affected by stage of lactation. J Nutr.

[bib1.bib3] Bofill-De Ros X, Gu S (2016). Guidelines for the optimal design of miRNA-based shRNAs. Methods.

[bib1.bib4] Bowman TA, O'Keeffe KR, D'Aquila T, Yan QW, Griffin JD, Killion EA, Salter DM, Mashek DG, Buhman KK, Greenberg AS (2016). Acyl CoA synthetase 5 (ACSL5) ablation in mice increases energy expenditure and insulin sensitivity and delays fat absorption. Mol Metab.

[bib1.bib5] Bronkhorst AW, van Cleef KW, Venselaar H, van Rij RP (2014). A dsRNA-binding protein of a complex invertebrate DNA virus suppresses the Drosophila RNAi response. Nucleic Acids Res.

[bib1.bib6] Bu SY, Mashek DG (2010). Hepatic long-chain acyl-CoA synthetase 5 mediates fatty acid channeling between anabolic and catabolic pathways. J Lipid Res.

[bib1.bib7] Catala-Rabasa A, Ndagire D, Sabio JM, Fedetz M, Matesanz F, Alcina A (2011). High ACSL5 transcript levels associate with systemic lupus erythematosus and apoptosis in Jurkat T lymphocytes and peripheral blood cells. PloS ONE.

[bib1.bib8] Fahy E, Subramaniam S, Murphy RC, Nishijima M, Raetz CR, Shimizu T, Spener F, van Meer G, Wakelam MJ, Dennis EA (2009). Update of the LIPID MAPS comprehensive classification system for lipids. J Lipid Res.

[bib1.bib9] Fahy E, Cotter D, Sud M, Subramaniam S (2011). Lipid classification, structures and tools. Biochim Biophys Acta.

[bib1.bib10] Graugnard DE, Piantoni P, Bionaz M, Berger LL, Faulkner DB, Loor JJ (2009). Adipogenic and energy metabolism gene networks in *longissimus lumborum* during rapid post-weaning growth in Angus and Angus x Simmental cattle fed high-starch or low-starch diets. BMC Genomics.

[bib1.bib11] Gulick AM, Starai VJ, Horswill AR, Homick KM, Escalante-Semerena JC (2003). The 1.75 A crystal structure of acetyl-CoA synthetase bound to adenosine-5
${}^{\prime }$
-propylphosphate and coenzyme A. Biochemistry.

[bib1.bib12] Li LO, Klett EL, Coleman RA (2010). Acyl-CoA synthesis, lipid metabolism and lipotoxicity. Biochim Biophys Acta.

[bib1.bib13] Lopes-Marques M, Cunha I, Reis-Henriques MA, Santos MM, Castro LF (2013). Diversity and history of the long-chain acyl-CoA synthetase (*Acsl*) gene family in vertebrates. BMC Evol Biol.

[bib1.bib14] Mashek DG, Bornfeldt KE, Coleman RA, Berger J, Bernlohr DA, Black P, DiRusso CC, Farber SA, Guo W, Hashimoto N, Khodiyar V, Kuypers FA, Maltais LJ, Nebert DW, Renieri A, Schaffer JE, Stahl A, Watkins PA, Vasiliou V, Yamamoto TT (2004). Revised nomenclature for the mammalian long-chain acyl-CoA synthetase gene family. J Lipid Res.

[bib1.bib15] Meller N, Morgan ME, Wong WP, Altemus JB, Sehayek E (2013). Targeting of Acyl-CoA synthetase 5 decreases jejunal fatty acid activation with no effect on dietary long-chain fatty acid absorption. Lipids Health Dis.

[bib1.bib16] Mendez R, Kollmorgen G, White MF, Rhoads RE (1997). Requirement of protein kinase C zeta for stimulation of- protein synthesis by insulin. Mol Cell Biol.

[bib1.bib17] Nelson GJ, Ackman RG (1988). Absorption and transport of fat in mammals with emphasis on n-3 polyunsaturated fatty acids. Lipids.

[bib1.bib18] Sheng Y, Ren H, Limbu SM, Sun Y, Qiao F, Zhai W, Du ZY, Zhang M (2018). The Presence or Absence of Intestinal Microbiota Affects Lipid Deposition and Related Genes Expression in Zebrafish (Danio rerio). Front Microbiol.

[bib1.bib19] Shmakov S, Abudayyeh OO, Makarova KS, Wolf YI, Gootenberg JS, Semenova E, Minakhin L, Joung J, Konermann S, Severinov K, Zhang F, Koonin EV (2015). Discovery and Functional Characterization of Diverse Class 2 CRISPR-Cas Systems. Mol Cell.

[bib1.bib20] Snead NM, Wu X, Li A, Cui Q, Sakurai K, Burnett JC, Rossi JJ (2013). Molecular basis for improved gene silencing by Dicer substrate interfering RNA compared with other siRNA variants. Nucleic Acids Res.

[bib1.bib21] Watkins PA, Maiguel D, Jia Z, Pevsner J (2007). Evidence for 26 distinct acyl-coenzyme A synthetase genes in the human genome. J Lipid Res.

[bib1.bib22] Xiao H, Zhao Z, Fang X, Yu H, Long X, Jiang P, Yang R (2016). Association of the Acsl5 Gene G.33185918g
>
A and G.33186348c
>
T Mutations with Carcass and Meat Quality Traits of Chinese Simmental-Cross Steers. J Anim Plant Sci.

[bib1.bib23] Yang TT, Cheng L, Kain SR (1996). Optimized codon usage and chromophore mutations provide enhanced sensitivity with the green fluorescent protein. Nucleic Acids Res.

[bib1.bib24] Zhang JW, Tang QQ, Vinson C, Lane MD (2004). Dominant-negative C/EBP disrupts mitotic clonal expansion and differentiation of 3T3-L1 preadipocytes. P Natl Acad Sci USA.

